# Impacto de la pandemia de la COVID-19 en la actividad de los servicios de urgencias de atención primaria: estudio comparativo entre periodos de 2019 y 2020

**DOI:** 10.1016/j.aprim.2023.102600

**Published:** 2023-03-01

**Authors:** Ángela Jiménez-García, Genoveva Pérez-Romero, César Hueso-Montoro, María Paz García-Caro, Luís Castro-Rosales, Rafael Montoya-Juárez

**Affiliations:** a Servicio de Urgencias de Atención Primaria de Distrito Sanitario Granada Metropolitano, Instituto Biosanitario Granada (IBS), Granada, España; b Departamento de Enfermería, Universidad de Jaén, Jaén, España; c Instituto Biosanitario Granada (IBS), Centro de Investigación Mente, Cerebro y Comportamiento, Universidad de Granada, Granada, España; d Departamento de Enfermería, Universidad de Granada, Granada, Spain; e Servicio de Estadística del Distrito Sanitario Granada Metropolitano, Granada, España

**Keywords:** COVID-19, SARS-CoV-2, Pandemias, Servicios de urgencias y emergencias, Triaje, Atención primaria de salud, COVID-19, SARS-CoV-2, Pandemics, Emergency Medical Services, Triage, Primary Health Care

## Abstract

**Objetivo:**

Comparar la atención prestada por los servicios de urgencias de atención primaria durante el confinamiento por la COVID-19 (marzo-junio de 2020) y el mismo periodo de 2019.

**Diseño:**

Estudio descriptivo retrospectivo.

**Emplazamiento:**

Zona básica de salud de la ciudad de Granada.

**Participantes:**

Diez mil setecientos noventa registros de urgencias, 3.319 en 2020 y 7.471 en 2019.

**Mediciones principales:**

Edad, sexo, servicio, franjas horarias, derivación al alta, niveles de prioridad, tiempos de espera, procesos previos y motivos de consulta. Se emplearon la «t» de Student y Chi-cuadrado para variables continuas y categóricas. Se calcularon el tamaño del efecto (d de Cohen) y OR junto con el IC al 95%.

**Resultados:**

Las urgencias disminuyeron en 2020 con respecto a 2019, aumentó el porcentaje de las urgencias prioridad v (p < 0,01), derivaciones al alta al domicilio (p = 0,01) y traslados al hospital (p < 0,01), en detrimento de las derivaciones a los médicos de familia (p < 0,01). Aumentaron en 2020 las urgencias en horario nocturno (p < 0,01) y en barrios de renta baja (p < 0,01). El tiempo de espera para clasificación disminuyó (p < 0,01), pero el total de asistencia aumentó en 2020 (p < 0,01). Los pacientes atendidos en 2020 fueron de mayor edad (p < 0,01) y con un mayor número de procesos previos (p < 0,01), destacando los pacientes con ansiedad, depresión o somatizaciones (p < 0,01) y diabetes (p = 0,041). Aumentaron las consultas relacionadas con diversos síntomas de la COVID-19, problemas de salud mental y enfermedades crónicas.

**Conclusiones:**

Los servicios de urgencias de atención primaria ofrecen ventajas adicionales ante situaciones como la pandemia de la COVID-19, dado que permiten canalizar parte de la demanda sanitaria.

## Abstract (en)

**Objective:**

To compare the care provided by primary care emergency services during the COVID19 lockdown (March-June 2020) and the same period in 2019.

**Design:**

Retrospective descriptive study.

**Setting:**

Basic Health Area of Granada.

**Population:**

10.790 emergency reports, 3.319 in 2020 and 7.471 in 2019.

**Outcomes:**

Age, sex, service, shifts, referrals, priority levels, care times, previous processes, and reasons for consultation. T-Student and Chi Square were used for continuous and categorical variables. Effect size (Cohen's d) and OR along with 95% CI were calculated.

**Results:**

The patients attended by primary care emergency services decreased in 2020 compared to 2019, but the percentage of Priority V cases (p < 0.01), home discharges (p = 0.01) and hospital transfers (p < 0.01) increased, and referrals to family doctors (p < 0.01) decreased. In 2020, the percentage of emergencies at night (p < 0.01) and in low-income neighborhoods (p < 0.01) increased. Waiting time for classification decreased (p < 0.01), but total care time increased in 2020 (p < 0.01). The patients seen in 2020 were older (p < .001), and with a greater number of previous processes (p < 0.01), highlighting patients with anxiety, depression, or somatization (p < 0.01) and diabetes (p = 0.041). Consultations related to various symptoms of COVID19, mental health problems and chronic pathologies increased.

**Conclusions:**

Primary care emergency services offer additional advantages in situations such as the COVID19 pandemic, as they allow channeling part of the health demand.

## Introducción

El 11 de marzo de 2020 la Organización Mundial de la Salud declaró la enfermedad causada por la transmisión del virus SARS-CoV-2 como pandemia de la COVID-19. El 14 de marzo el gobierno español declaró el «estado de alarma» en todo el territorio nacional y decretó el confinamiento domiciliario de toda la población que no ejerciera actividades no esenciales.

La saturación de los centros sanitarios se hizo evidente, siendo los servicios de urgencias unos de los que más sufrieron estos efectos. Se redistribuyeron los medios humanos disponibles, se remodelaron los circuitos de atención y se reorganizaron los espacios para hacer frente a la COVID-19^1^.

En España esta adaptación dependió en gran medida de las administraciones regionales con competencias sanitarias transferidas. Andalucía es una de las comunidades autónomas más extensas y pobladas de España, situada en el sur, con una población de 8.494.155 habitantes^2^ (a 1 de enero de 2022), distribuida en 8 provincias, entre las que se encuentra Granada.

En Andalucía las urgencias están conformadas por los servicios de urgencias hospitalarios y los servicios de urgencias de atención primaria (SUAP)^3^. Los SUAP están organizados por distritos sanitarios de atención primaria y se caracterizan por ofrecer atención a la población a través de un equipo móvil en el propio domicilio del paciente o en el punto fijo de urgencias, en el horario en el que el centro de atención primaria está cerrado, de 15:00 h de la tarde hasta las 8:00 h de la mañana los días laborales, así como sábados, domingos y festivos las 24 horas. Disponen de protocolos asistenciales para la atención de procesos agudos tiempo-dependientes, clasificación avanzada^4^ y equipos móviles de cuidados avanzados^5^.

Algunos estudios han analizado el impacto de la pandemia de la COVID-19 sobre los servicios de urgencias a nivel internacional^6^, ^7^, ^8^. En nuestro país los estudios publicados se han centrado en las urgencias atendidas en el ámbito hospitalario^9^, ^10^, ^11^. Sin embargo, hasta ahora no se ha analizado el comportamiento de los usuarios en los SUAP durante la pandemia. Esto supone una importante laguna, puesto que toda la atención primaria en Europa se vio afectada durante la pandemia^12^, y debe contemplarse el uso de los SUAP como un recurso alternativo, tanto para la demanda urgente como para la demanda sanitaria a la que habitualmente da respuesta atención primaria.

Por otro lado, aunque se han realizado estudios de la evolución en la demanda de enfermedades concretas, como la EPOC^13^, la insuficiencia cardiaca^14^ o la enfermedad psiquiátrica^15^, este estudio compara la demanda asistencial, el comportamiento y perfil de los usuarios que acudieron a los SUAP durante el confinamiento por la COVID-19 (14 de marzo de 2020-21 de junio de 2020) y el mismo periodo de 2019.

## Método

Se realizó un estudio descriptivo retrospectivo de las urgencias atendidas en los SUAP de la zona básica de salud de la ciudad de Granada integrada por 3 centros con SUAP: La Chana, Zaidín y Gran Capitán. Las zonas a las que dan cobertura los distintos SUAP de Granada son heterogéneas, tanto por su extensión como por las características de la población que atienden, todos ellos incluyen tanto distritos de la ciudad como pueblos del cinturón o área metropolitana. Así, el SUAP Chana atiende a población de 3 distritos municipales (Norte, Beiro y Chana), 2 de ellos con rentas medias bajas (Norte: 20.354 € y Chana: 23.291 €), el SUAP Zaidín a 3 distritos (Ronda, Genil y Zaidín), 2 de ellos con rentas medias altas (Genil: 30.181 €, Ronda: 30.703 €), y el SUAP Gran Capitán a 2 distritos (Centro y Albayzín), con una renta media de 34.848 € en la zona Centro, según datos de la Agencia Tributaria de 2017.

Se incluyeron urgencias de pacientes de todas las edades atendidos durante el estado de alarma (14 de marzo de 2020 a 21 de junio de 2020) y el mismo periodo del año anterior. Se excluyeron asistencias que estuvieran incompletas (ausencia de datos en alguna de las variables de estudio).

Todos los episodios fueron recogidos en una base de datos propia tras volcar de forma automática los datos anonimizados proporcionados por el Servicio Andaluz de Salud, atendiendo a las variables del estudio:

∘ Edad: categorizada en 3 grupos: < 14 años, 15-65 y > 65 años.
∘ Sexo.
∘ SUAP: la Chana, Zaidín y Gran Capitán.
∘ Franjas horarias de atención: de lunes a viernes: tarde (15:00 a 22:00 h) y noche (22:00 a 8:00 h). Sábados, domingos y festivos: mañana (8:00 a 15:00 h), tarde y noche.
∘ Derivación al alta: domicilio, médico de atención primaria y traslado al hospital.
∘ Niveles de prioridad según el sistema español de triaje: prioridad i a v.
∘ Tiempo de espera de clasificación (TECLA): periodo de tiempo expresado en minutos que trascurre desde el cierre del procedimiento administrativo de admisión hasta el inicio del triaje.
∘ Tiempo de clasificación (TICLA): periodo de tiempo expresado en minutos que trascurre desde el inicio del triaje hasta el cierre de este. Incluye la asignación del nivel de prioridad y del circuito asistencial y facultativo que asistirá al paciente.
∘ Tiempo asistencial total en el área de consultas (TATCO): periodo de tiempo expresado en minutos que transcurre desde que el paciente es admitido hasta la derivación.
∘ Procesos asistenciales integrados (PAI): episodios de urgencias en los que se atendió a pacientes previamente incluidos en los distintos PAI; el número total de procesos que presentaban los pacientes atendidos, así como pacientes cuyo PAI indicaba algún proceso crónico (cuidados paliativos, demencias, diabetes, EPOC o insuficiencia cardiaca).
∘ Motivo de consulta: corresponde a 179 síntomas que vienen predeterminados en el programa informático DIRAYA, empleado en los SUAP. Para facilitar el análisis se agruparon según la codificación CIE9, si bien algunos fueron tratados de modo independiente por ubicarse en varias categorías de este sistema de clasificación.

### Análisis de los datos

Se calcularon medidas de tendencia central y dispersión para describir variables continuas, así como frecuencia y porcentaje para las categóricas. Para la comparación entre los 2 periodos de estudio se utilizó la prueba «t» de Student para muestras independientes en el caso de variables continuas y la Chi cuadrado para variables categóricas. Se consideró que las variables siguen una distribución normal de acuerdo con el tamaño muestral y el teorema central del límite. Adicionalmente se calculó la d de Cohen y la OR (IC 95%) de prevalencia. Se consideró un nivel de significación estadística de 0,05. El análisis se realizó con el software IBM's SPSS© v.26.

## Resultados

**Figura 1:**
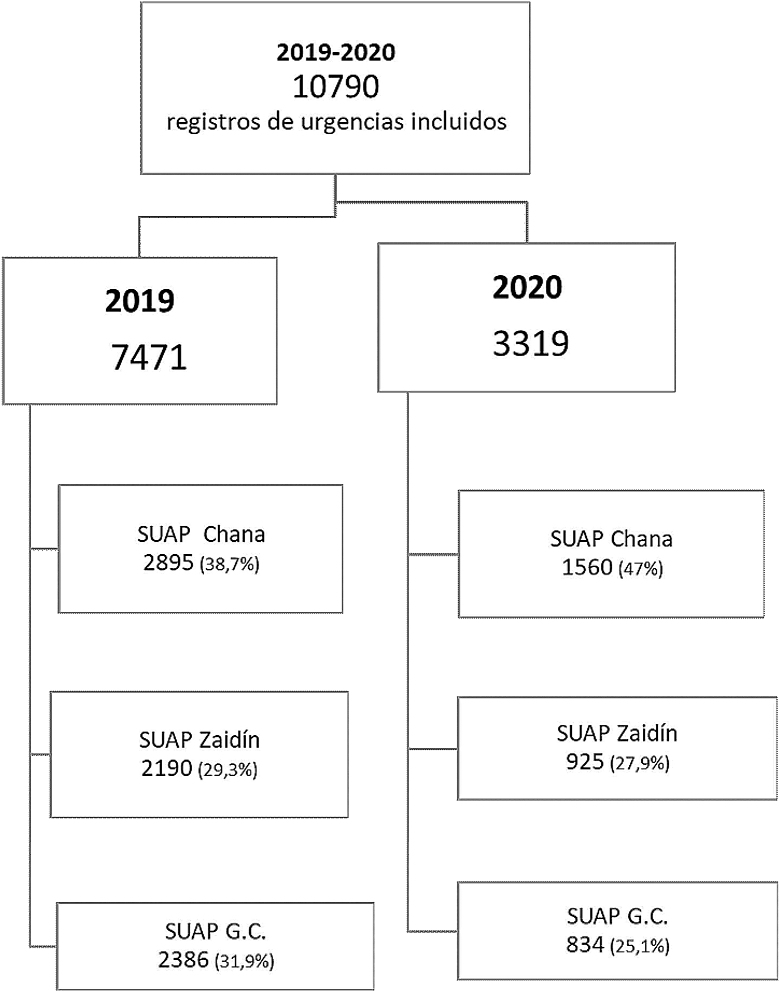
Esquema de estudio. Número total de registros en 2019 = 32.555 (SUAP Chana = 14.214; SUAP Gran Capitán = 9.368; SUAP Zaidín = 8973). Número total de registros en 2020 = 10.756 (SUAP Chana = 4.964; SUAP Gran Capitán = 2.362; SUAP Zaidín = 3.430).

Se analizaron 10.790 registros de urgencias entre ambos años, 3.319 (30,8%) en 2020 y 7.471 (69,2%) en 2019 (fig. 1). La diferencia en el número de registros fue de 4.152 (38,4%; IC 95% = 37,6-39,4%).

La media de edad de los pacientes atendidos fue superior en 2020 (43,28 ± 20,92; Me = 41; Mo = 21) con respecto a 2019 (38,72 ± 20,66; Me = 35; Mo = 20) (p < 0,01), con una proporción de menores de 14 años inferior (3,9% y 7,7%; p < 0,01; OR = 0,487, IC 95% = 0,400-0,592) y un aumento de mayores de 65 años (16,8% y 12,8%; p < 0,01; OR = 1,376, IC 95% = 1,228-1,542) en 2020. En 2019 fueron atendidos 2.902 hombres (38,8%) y 4.569 (61,2%) mujeres; mientras que en 2020 fueron 1.337 (40,3%) y 1.982 (59,7%), respectivamente. No existieron diferencias significativas con respecto al sexo entre ambos años.

Se observaron diferencias significativas con respecto a la prioridad de las urgencias (tabla 1), observándose que aumentó la proporción de urgencias identificadas como prioridad v y prioridad ii en 2020 con respecto a 2019 (p < 0,01).

**Tabla 1:** Niveles de prioridad y derivaciones al alta. Comparativa entre 2019 y 2020

	Año		p	
	2019	2020		OR (IC 95%)
*Prioridad*				
Prioridad I	2 (0,0%)	4 (0,1%)	0,057	NS
Prioridad II	43 (0,6%)	36 (1,1%)	0,004	1,894 (1,214-2,955)
Prioridad III	547 (7,3%)	153 (4,6%)	< 0,01	0,612 (0,509-0,735)
Prioridad IV	2902 (38,8%)	1020 (30,7%)	< 0,01	0,699 (0,640-0,762)
Prioridad V	3977 (53,2%)	2106 (63,5%)	< 0,01	1,525 (1,402-1,659)
*Derivaciones al alta*				
Domicilio	5470 (73,2%)	2527 (76,1%)	0,001	1,167 (1,062-1,283)
Médico de familia	1784 (23,9%)	677 (20,4%)	< 0,01	0,817 (0,739-0,903)
Traslado al hospital	135 (1,8%)	81 (2,4%)	0,030	1,359 (1,029-1,796)

Respecto a la derivación al alta se hallaron diferencias significativas entre ambos años en las 3 derivaciones observadas (tabla 1).

También se encontraron diferencias significativas entre los distintos centros estudiados y las franjas horarias de atención (tabla 2).

**Tabla 2:** Demanda asistencial en los distintos centros y franjas horarias. Comparativa entre 2019 y 2020

	Año	N (%)	p	OR (IC 95%)
*Centros*				
SUAP Chana	2019	2895 (38,7)	< 0,01	1,402 (1,291-1,522)
	2020	1560 (47,0)		
SUAP Zaidín	2019	2190 (29,3)	0,127	NS
	2020	925 (27,9)		
SUAP Gran Capitán	2019	2386 (31,9)	< 0,01	0,715 (0,652-0,784)
	2020	834 (25,1)		
*Franjas horarias*				
Mañana	2019	1486 (70,7)	0,107	NS
	2020	616 (29,3)		
Tarde	2019	4499 (60,2)	0,057	NS
	2020	1934 (58,3)		
Noche	2019	1486 (19,9)	< 0,01	1,215 (1,101-1,341)
	2020	769 (23,2)		

Los tiempos de atención variaron de forma diferente (tabla 3), la media del TECLA disminuyó en 2020 con respecto a 2019 (p < 0,01), la del TATCO aumentó (p < 0,01) y la del TICLA no fue significativa.

**Tabla 3:** Tiempos de atención en las urgencias. Comparativa entre 2019 y 2020

	Año	N	Media	SD	p	d
Tiempo de espera de clasificación (TECLA) (min)	2019	7.471	19,8231	26,241	< 0,01	0,133
	2020	3.319	14,2031	64,754		
Tiempo de clasificación (TICLA) (min)	2019	7.471	0,91358	3,911	0,362	NS
	2020	3.319	1,11678	18,354		
Tiempo de asistencia total en el área de consultas (TATCO) (min)	2019	7.471	36,2395	73,899	< 0,01	0,100
	2020	3.319	50,8696	237,115		

La proporción de pacientes incluidos en los PAI ansiedad, depresión, somatizaciones (p < 0,01) y diabetes (p = 0,041) aumentaron en 2020. Sin embargo, la proporción de pacientes con algún PAI de las enfermedades crónicas analizadas fue similar en los 2 periodos (tabla 4).

**Tabla 4:** Pacientes atendidos identificados con algún proceso asistencial integrado

	Año		p	
	2019	2020		OR (IC 95%)
Ansiedad, depresión, somatizaciones	123 (1,6%)	94 (2,8%)	< 0,01	1,741 (1,327-1,285)
Atención temprana	21 (0,3%)	6 (0,2%)	0,336	NS
Cáncer de cérvix/útero	1.377 (18,4%)	643 (19,4%)	0,247	NS
Cáncer de mama	296 (4,0%)	115 (3,5%)	0,213	NS
Cefaleas	188 (2,5%)	85 (2,6%)	0,892	NS
Cuidados paliativos	0 (0%)	1 (0%)	0,134	NS
Demencias	11 (0,1%)	3 (0,1%)	0,449	NS
Diabetes	292 (3,9%)	158 (4,8%)	0,041	1,229 (1,008-1,498)
Embarazo	30 (0,4%)	18 (0,5%)	0,311	NS
EPOC	126 (1,7%)	44 (1,3%)	0,165	NS
HBP y Ca. próstata	168 (2,2%)	73 (2,2%)	0,873	NS
Insuficiencia cardíaca	26 (0,3%)	20 (0,6%)	0,061	NS
Total procesos crónicos Cuidados paliativos + demencias + diabetes + EPOC + insuficiencia cardíaca	448 (6,0%)	222 (6,7%)	0,169	NS

Finalmente, se observaron diferencias significativas en la mayoría de los motivos de consulta entre los años 2019 y 2020 (tabla 5).

**Tabla 5:** Motivo de consulta. Comparativa entre 2019 y 2020

	Año		P	
	2019	2020		OR (IC 95%)
Enfermedades endocrinas, nutricionales y metabólicas	19 (0,3%)	13 (0,4%)	0,226	NS
Trastornos mentales, del comportamiento y del desarrollo neurológico	268 (3,6%)	163 (4,9%)	0,001	1,388 (1,137-1,694)
Enfermedades del ojo y sus anexos	200 (2,7%)	87 (2,6%)	0,868	NS
Enfermedades del oído y de la apófisis mastoides	160 (2,1%)	70 (2,1%)	0,914	NS
Enfermedades del aparato circulatorio	135 (1,8%)	91 (2,7%)	0,002	1,532 (1,171-2,005)
Enfermedades del aparato respiratorio (sin disnea)	1.076 (14,4%)	212 (6,4%)	< 0,01	0,406 (0,348-0,473)
Enfermedades del aparato digestivo	887 (11,9%)	482 (14,5%)	< 0,01	1,261 (1,119-1,421)
Enfermedades de la piel y del tejido subcutáneo	847 (11,3%)	550 (16,6%)	< 0,01	1,553 (1,383-1,745)
Enfermedades del aparato musculoesquelético y del tejido conectivo	733 (9,8%)	382 (11,5%)	0,007	1,196 (1,049-1,363)
Enfermedades del aparato genitourinario	943 (12,6%)	529 (15,9%)	< 0,01	1,313 (1,170-1,473)
Lesiones traumáticas, envenenamientos y otras consecuencias de causas externas	11 (0,1%)	5 (0,2%)	0,966	NS
Disnea	109 (1,5%)	77 (2,3%)	0,002	1,604 (1,195-2,154)
Mareo	147 (2,0%)	83 (2,5%)	0,077	NS
Malestar general	1.100 (14,7%)	306 (9,2%)	< 0,01	0,588 (0,515-0,672)
Fiebre	680 (9,1%)	171 (5,2%)	< 0,01	0,542 (0,456-0,645)

## Discusión

Los resultados de este estudio muestran que las urgencias atendidas por los SUAP disminuyeron durante el confinamiento por la COVID-19 con respecto al mismo periodo de 2019, aunque este descenso fue heterogéneo con respecto a los niveles de prioridad, las derivaciones, los horarios y los centros. También muestran que en 2020 disminuyó el TECLA pero aumentó el TATCO con respecto a 2019.

El perfil de los pacientes atendidos en 2020 fue de mayor edad, y aumentaron las urgencias de sintomatología compatible con la COVID-19, los problemas de salud mental y las enfermedades crónicas.

Diversos estudios han señalado que hubo una disminución de la atención urgente durante las primeras olas de la pandemia^11^, ^16^, ^17^, sin embargo, este es el primer estudio que ha identificado esta reducción en SUAP. Los motivos que han señalado otros autores son el miedo de la población, la novedosa situación de confinamiento y que las necesidades banales, o incluso urgentes, pasaron a segundo plano con respecto a los síntomas graves compatibles con la COVID-19^16^.

La disminución de la atención urgente en los SUAP afectó a los niveles de prioridad de forma heterogénea. En situación de normalidad la prioridad v (situaciones no prioritarias) suele ocupar el mayor porcentaje de demandas, y era de esperar que durante el confinamiento disminuyeran, por los motivos anteriormente citados. Sin embargo, en nuestro estudio el porcentaje aumentó en 2020 con respecto a 2019. Una hipótesis que puede explicar este hecho es que los usuarios emplearan los SUAP como acceso al sistema sanitario para realizar trámites administrativos, prescripción de medicamentos, o problemas poco urgentes pero necesarios de resolver, dadas las limitaciones de acceso a los centros de salud durante gran parte del periodo estudiado en 2020.

Diferentes estudios han señalado las enormes dificultades soportadas en el ámbito extrahospitalario durante este tiempo, dando lugar a una atención insuficiente a pacientes en situación de enorme vulnerabilidad^18^, ^19^. El acceso limitado a estos centros dio lugar a la disminución de la asistencia o insuficiente seguimiento de pacientes crónicos o ancianos^20^. También provocó insatisfacción y sentimiento de abandono de muchos usuarios con pocos recursos o importantes deficiencias informáticas o comunicativas, que se vieron obligados a utilizar la consulta telefónica o telemática^21^, ^22^, ^23^. Parece coherente la disminución observada de las derivaciones al alta al médico de familia en 2020 frente a las ocurridas en 2019, así como el ascenso relativo de las derivaciones a domicilio y al hospital.

Uno de los hallazgos más llamativos de este estudio fue la distribución asimétrica en el porcentaje de urgencias atendidas en los distintos SUAP (disminuyó en Gran Capitán y aumentó en La Chana), y en las distintas franjas horarias (aumentaron las urgencias nocturnas). Cabe resaltar que las zonas atendidas por cada SUAP presentan distintas características demográficas y socioeconómicas. Mientras La Chana es un barrio con una renta media por hogar de 18.975 €, en Gran Capitán era de 34.843 €, según datos de 2019^24^. En Gran Capitán la población que predomina son personas mayores y estudiantes universitarios (asistencia por desplazamiento temporal). Una hipótesis que pueda explicar las diferencias entre barrios es que las personas mayores confinadas en sus domicilios recurrieron menos a las urgencias y los estudiantes universitarios volvieron a sus lugares de origen, mientras que los trabajadores y empleados en trabajos esenciales continuaron activos, utilizando los SUAP en horario nocturno, tras la jornada laboral.

Respecto a los tiempos de atención el TECLA disminuyó en 2020, lo que puede indicar que las situaciones atendidas por los SUAP fueron fácilmente clasificables respecto a la prioridad. En cambio, el TATCO aumentó. Esto puede estar relacionado con situaciones inusitadas para los profesionales, como la colocación y retirada del EPI en cada asistencia. No se ha encontrado referencias con respecto a esta situación más allá de las alusiones a la relevancia y el rigor necesario para ejecutar estas medidas, y que el tiempo necesario dependía de la pericia y experiencia del profesional.

Con respecto a los motivos de consulta se observó un aumento de las consultas por disnea, enfermedades del aparato circulatorio, digestivo, de la piel y del tejido subcutáneo, musculoesquelético y genitourinario. La disnea fue uno de los principales síntomas identificados de la COVID-19, lo que puede explicar el aumento en el porcentaje de pacientes que consultaron por este síntoma en 2020. Sin embargo, con el paso de los meses también se tipificaron como síntomas de la COVID-19 los digestivos, como diarrea, o cutáneos como el exantema, lo cual puede explicar también el aumento de este tipo de síntomas. Otros como los circulatorios, musculoesqueléticos y genitourinarios, en cambio, no se explican solo por la incidencia de la COVID-19, y parecen más relacionados con el mayor uso de los SUAP por pacientes crónicos para el seguimiento de sus enfermedades o por descompensaciones. Esto puede explicar el aumento del porcentaje de pacientes atendidos con el PAI «diabetes», el aumento de la edad y el número medio de PAI de la población atendida en urgencias en 2020. También es coherente con esta hipótesis el drástico descenso del porcentaje de pacientes atendidos cuyo principal motivo de consulta fue «malestar general». Parece que los usuarios prefirieron no consultar a los SUAP sin un motivo claro de consulta, bien por miedo o por responsabilidad ante una atención primaria colapsada^12^.

Por último, cabe destacar el aumento del porcentaje de pacientes que demandaron atención en 2020 incluidos en el PAI de «ansiedad, depresión, somatizaciones», en consonancia con el aumento de la proporción de pacientes cuyo motivo de consulta fue «trastornos mentales, del comportamiento y del desarrollo neurológico». Varios estudios han señalado efectos psicológicos a corto plazo de la pandemia identificados en la población general como un aumento del malestar psicológico, la ansiedad, el miedo, los síntomas depresivos, la sintomatología de estrés postraumático, el insomnio, el estigma, la marginalidad y el prejuicio hacia la enfermedad, así como una disminución de las emociones positivas^25^, ^26^, ^27^.

Este estudio tiene limitaciones para generalizar sus resultados, ya que se trata de un estudio observacional retrospectivo de un solo distrito sanitario, y cada zona tiene su idiosincrasia demográfica y organización sanitaria.

Los resultados de este estudio indican que las urgencias atendidas por los SUAP disminuyeron en 2020 con respecto a 2019, aunque aumentó el porcentaje de las urgencias prioridad v, derivaciones al alta al domicilio y traslados al hospital, en detrimento de las derivaciones a los médicos de familia. Aumentaron en 2020 las urgencias en horario nocturno y en barrios de renta baja. El tiempo de espera para clasificación disminuyó, pero el total de asistencia aumentó en 2020. Los pacientes atendidos en 2020 fueron de mayor edad y con un mayor número de procesos previos, destacando los pacientes con síntomas compatibles con COVID, problemas de salud mental y enfermedades crónicas. Los SUAP ofrecen ventajas adicionales ante situaciones como la pandemia por la COVID-19, dado que permiten canalizar parte de la demanda sanitaria. No obstante, es necesario confirmar esta hipótesis, comparando la dinámica en la atención urgente en aquellos lugares en los que no se dispone de estos dispositivos.

**Lo conocido sobre el tema**

- La saturación de los servicios de urgencias se hizo evidente durante el estado de alarma en España debido a la pandemia por la COVID-19.
- Se ha estudiado el impacto de la pandemia en los servicios de urgencias en los ámbitos internacional y nacional, principalmente en el entorno hospitalario.
- Es necesario conocer el impacto en otros servicios de urgencias, como es el caso del SUAP.

**Qué aporta este estudio**

- Las urgencias atendidas por el SUAP disminuyeron durante el confinamiento por la COVID-19, si bien esta disminución fue heterogénea.
- Los SUAP permitieron canalizar parte de la demanda sanitaria condicionada por las restricciones de acceso de los centros de salud durante la pandemia.
- Los SUAP suponen un valor añadido en la atención sanitaria ante situaciones como la pandemia de la COVID-19.

## Consideraciones éticas

Estudio aprobado por el Comité de Ética de la Investigación de Andalucía el 11 enero de 2021 con el código EPA_SUAP.

## Financiación

Los autores declaran la no existencia de financiación en relación con el presente artículo.

## Conflicto de intereses

Los autores declaran no tener conflicto de intereses en relación con el presente artículo.
